# Missouri Valley

**Published:** 1899-08

**Authors:** Jas. S. Kelly

**Affiliations:** Secretary


Secretary.

### MISSOURI VALLEY VETERINARY MEDICAL ASSOCIATION.

THE annual meeting of this Association was held at the Y. M. C. A. Building, in the city of St. Joseph, Missouri, on the 26th day of June, 1899. The meeting was convened at 8 P.M., with Dr. S. E. Bennett, President, in the chair. Dr. Kelly, in the absence of Dr. W. A. Heck, Secretary of the Association, acted as secretary of the meeting. Those in attendance were: Drs. Bennett, Stewart, and Moore, Kansas City; W. N. Hobbs, Holton, Kan.; Forbes, Kelly, Washburn, Wright, and Netherton, of St. Joseph. Visitors and others present: Drs. James Wilson, J. E. Blackwell, Thomas H. Ripley, Joseph Good, H. G. Patterson, John A. Sloan, Joseph Thackaberry, St. Joseph; Dr. A. S. Peters, of Lincoln, Neb.; Dr. V. A. Schaefer, of Tekamah, Neb.; L. C. Brown, of Hamilton, Mo.; M. Y. Schaefer, of Mount Ayr, Iowa; G. R. Conrad, Sabetha, Kan., and Dr. Hansen.

Applications of new members were in order.

The Board of Censors reported the following applications of new members: G. R. Conrad, L. D. Brown, H. G. Patterson, V. A. Schaefer, M. Y. Schaefer, John A. Sloan, and Joseph Thackaberry. The applicants were found to be eligible to membership and satisfactory to the Association. Moved by Dr. Forbes that the rules of the Association be suspended, and that the Secretary be instructed to cast the vote of the Association for the above-named applicants for membership; seconded and carried. The Secretary cast the ballot of the Association and the candidates were duly elected.

Next in order was the reading of the report of the Secretary. It was moved and seconded that the report of the Secretary be received as read and placed on file; motion carried.

The Secretary then read the resignations of Drs. W. A. Heck, W. P. Steddom, and W. P. McCurdy. It was moved by Dr. Forbes that the resignations be accepted, and in the case of Dr. Heck he suggested that as he had proven an efficient member and able officer of the Association while acting in the capacity of Secretary, it would be a graceful act on the part of the Association to elect him an honorary member; motion to accept the resignations of Drs. Steddom and McCurdy carried.

The next business was the election of officers. Nominations first in order for the office of President. Dr. Forbes: Seeing that the Association has been so flourishing during the past year under the present executive, I think another term desirable, and I propose the name of Dr. Bennett for re-election for another year. Dr. Bennett: I appreciate the courtesy, gentlemen, of re-electing me as President for another term, but I do not think the flourishing condition is due in any manner to my efforts, but to those of the Secretary. What we want is a hustling Secretary, and I think I can do more for the Association as a private member and not as President, and I prefer not to serve another year. Dr. Stewart: I propose the name of Dr. Forbes, who has been an active member of the Association. Dr. Forbes: It is probable that some of the other offices will be filled from St. Joseph, and I think it would be advisable to divide the offices between the other places on the river, and would withdraw my name. Balloting upon the nominations of Drs. Bennett and Forbes now in order. The President thereupon appointed Drs. Stewart and Washburn tellers to receive and count the ballots cast. A ballot being taken, the tellers announced that ten votes had been cast for Dr. Forbes and five votes for Dr. Bennett. Dr. Bennett: According to the ballots cast by the Association Dr. Forbes has been duly elected President of the Association for the ensuing year.

Dr. Moore presented next the name of Dr. Bennett for First Vice-President. There being but one nominee, the rules were suspended and the Secretary instructed to cast the ballot of the Asscoiation, and Dr. Bennett was unanimously elected First Vice-President.

Dr. Stewart presented the name of Dr. V. A. Schaefer for the office of

Second Vice-President, and there being but one nomination, the rules were suspended and the Secretary instructed to cast the ballot of the Association, Dr. Schaefer being unanimously elected Second Vice-President.

The name of Dr. James S. Kelly was presented by Dr. Forbes for the office of Secretary and Treasurer. It was moved and seconded that the President cast the ballot of the Association for Dr. Kelly as Secretary and Treasurer for the ensuing year. The motion prevailed, and Dr. Kelly was elected.

Drs. Washburn, Netherton, Bennett, Moore, and Stewart were elected as the Board of Censors for the coming year.

Dr. Bennett then vacated the chair and said: I thank the Association for the courtesy extended to me during the past year, and I hope the members will take as much interest in the Association during the coming year as they have in the past. I now turn the office over to Dr. Forbes.

Dr. Forbes, on assuming the Presidency, said: As the Association has insisted, I will assume the duties of President, and I hope I will be able to conduct the affairs of the Association in the same efficient manner that they have been conducted during the past year. I crave the indulgence of the members, and hope that any fault that may occur will be overlooked.

A very entertaining and instructive clinic was held during the day at the office of Dr. E. J. Netherton, with the able assistance of Dr. H. G. Patterson. The Association was furnished with clinic material in abundance; the veterinarians of St. Joseph worked faithfully for the success of the meeting, and nothing was left undone to make it one of the most interesting gatherings which the society has yet enjoyed. At the close of the literary part of the programme the society adjourned to the Hotel Donovan, where a banquet had been prepared, and spent the remaining time responding to toasts and having a generally good time, until the small hours of the night, after which adjournment took place, to meet in Kansas City in October.

JAS. S. KELLY,
Secretary.

